# Six-month contingency management effects on smoking cessation in individuals with substance use disorders

**DOI:** 10.1192/j.eurpsy.2021.2172

**Published:** 2021-08-13

**Authors:** G. Aonso-Diego, A. Krotter, Á. García-Pérez, R. Secades-Villa

**Affiliations:** Department Of Psychology, University of Oviedo, Oviedo, Spain

**Keywords:** contingency management, smoking cessation, Substance Use Disorder, effectiveness

## Abstract

**Introduction:**

Persons with substance use disorders (SUD) smoke at strikingly high rates and tobacco use cessation rates are notably low in this population. Contingency Management (CM) is effective to promote substance abstinence, including tobacco, in a large range of populations. CM is scarcely implemented in clinical settings mainly due to barriers at the therapist and organizational levels.

**Objectives:**

The study sought to examine the additive effectiveness of CM on Cognitive-Behavioral Therapy (CBT) over long-term smoking abstinence in persons undergoing SUD treatment.

**Methods:**

A total of 54 smokers (75.9% males, M_age_=46.19, SD=9.21) were randomly assigned to CBT (n=30) or to CBT+CM (n=24). Interventions consisted of eight weeks of group-based therapy. Participants were instructed to gradually reduce their nicotine intake by 20% weekly. The CM arm was voucher-based, and the primary outcome was biochemically verified tobacco abstinence (CO≤4ppm, and urine cotinine≤80ng/ml).

**Results:**

A total of 42/54 (77.78%) participants completed the treatment (73.33% in CBT and 83.33% in CBT+CM; p=.380). At the end of treatment, participants in CBT+CM showed higher 24-hour smoking abstinence (50% vs. 20%, p=.032); however, both treatment conditions show equal abstinence rates in the remaining follow-ups (CBT_1month_= 13.33% vs. CBT+CM_1month_= 25%; CBT_2months_= 10% vs. CBT+CM_2months_= 16.66%; CBT_3months_= 10% vs. CBT+CM_3months_= 16.66%; CBT_6months_= 10% vs. CBT+CM_6months_= 8.33%; all p-values ≥ .244).

**Conclusions:**

CM facilitates early abstinence outcomes in smokers with SUD more than CBT only does. However, no additive effects of CM were observed at long-term, suggesting the convenience to intensify CM schedules or using technology platforms for incentives delivery.

**Disclosure:**

No significant relationships.

